# Insights From Y-STRs: Forensic Characteristics, Genetic Affinities, and Linguistic Classifications of Guangdong Hakka and She Groups

**DOI:** 10.3389/fgene.2021.676917

**Published:** 2021-05-24

**Authors:** Chunfang Luo, Lizhong Duan, Yanning Li, Qiqian Xie, Lingxiang Wang, Kai Ru, Shahid Nazir, Muhammad Jawad, Yifeng Zhao, Fenfen Wang, Zhengming Du, Dehua Peng, Shao-Qing Wen, Pingming Qiu, Haoliang Fan

**Affiliations:** ^1^School of Forensic Medicine, Southern Medical University, Guangzhou, China; ^2^Heyuan Municipal Public Security Bureau, Heyuan, China; ^3^Beijing Municipal Public Security Bureau, Beijing, China; ^4^School of Basic Medicine, Gannan Medical University, Ganzhou, China; ^5^Institute of Archaeological Science, Fudan University, Shanghai, China; ^6^Department of Forensic Sciences, University of Health Sciences, Lahore, Pakistan; ^7^Nanjing Zhenghong Judicial Identification Institute, Nanjing, China; ^8^First Clinical Medical College, Hainan Medical University, Haikou, China; ^9^School of Basic Medicine and Life Science, Hainan Medical University, Haikou, China

**Keywords:** Guangdong Hakka, Guangdong She, Y-STR, forensic characteristics, phylogenetic analyses

## Abstract

Guangdong province is situated in the south of China with a population size of 113.46 million. Hakka is officially recognized as a branch of Han Chinese, and She is the official minority group in mainland China. There are approximately 25 million Hakka people who mainly live in the East and North regions of China, while there are only 0.7 million She people. The genetic characterization and forensic parameters of these two groups are poorly defined (She) or still need to be explored (Hakka). In this study, we have genotyped 475 unrelated Guangdong males (260 Hakka and 215 She) with Promega PowerPlex^®^ Y23 System. A total of 176 and 155 different alleles were observed across all 23 Y-STRs for Guangdong Hakka (with a range of allele frequencies from 0.0038 to 0.7423) and Guangdong She (0.0047–0.8605), respectively. The gene diversity ranged from 0.4877 to 0.9671 (Guangdong Hakka) and 0.3277–0.9526 (Guangdong She), while the haplotype diversities were 0.9994 and 0.9939 for Guangdong Hakka and Guangdong She, with discrimination capacity values of 0.8885 and 0.5674, respectively. With reference to geographical and linguistic scales, the phylogenetic analyses showed us that Guangdong Hakka has a close relationship with Southern Han, and the genetic pool of Guangdong Hakka was influenced by surrounding Han populations. The predominant haplogroups of the Guangdong She group were O2-M122 and O2a2a1a2-M7, while Guangdong She clustered with other Tibeto-Burman language-speaking populations (Guizhou Tujia and Hunan Tujia), which shows us that the Guangdong She group is one of the branches of Tibeto-Burman populations and the Huonie dialect of She languages may be a branch of Tibeto-Burman language families.

## Introduction

**FIGURE 1:**
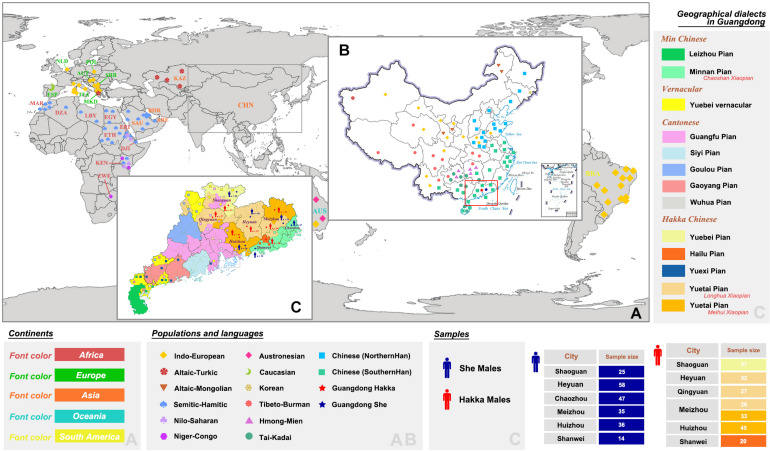
Locations of population distributions and sampling information. **(A,B)** Geographic positions of Guangdong Hakka, Guangdong She, and 159 worldwide populations with nine language families (48,637 individuals in total). **(C)** Distributions of geographical dialects in Guangdong province and detailed sampling information of Guangdong Hakka and Guangdong She groups.

Hakka is one of the far-reaching ethnic groups that have a worldwide distribution and is officially recognized as a branch of Han Chinese in China. Hakka is a unique group that is not named after the region (Zhong, 2019). There are about 80 million Hakka people, and ∼50 million are situated in southern parts of China (mainly including Guangdong, Jiangxi, Fujian, Guangxi, Sichuan, Hainan, Hunan, Zhejiang, Taiwan, Hongkong, and Macao). Guangdong province is an important region for Lingnan culture, which has its unique styles of language, history, and culture and which lies in the southernmost part of mainland China. The Hakka population is mainly settled in the eastern and northern regions of Guangdong, comprising Meizhou, Heyuan, Huizhou, Shaoguan, and Qingyuan (Figure 1). The origin of Hakka has not been clearly defined yet. At present, there are two views about the origin of the Hakka population, either they belong to Northern Han (Luo, 1989) or they belong to Southern Han (Fang, 2007). According to a previous study (Li et al., 2003), majority of the Fujian Hakka gene pool (80.2%) came from Northern Han based on 14 Y-SNPs. On the other hand, the frequency of a 9-bp deletion in mitochondrial region V is 21.74% in Meizhou Hakka, which indicated that Meizhou Hakka had close relationships with Fujian Hakka (∼0.197) and other populations from South China (Cai et al., 2015). From the perspective of physical anthropology, Zheng et al. found that the physical characteristics of the Hakka population are a mixture of South-Asian and North-Asian ethnic populations, which is determined by 86 anthropologic characteristics in 650 male and 704 female Chinese Hakka adults living in Guangdong and Jiangxi (Zheng et al., 2013). Guangdong province is home to the Hakka population, but there is no comprehensive study available on the Y-chromosomal prospect of Guangdong Hakka.

She is one of the largest ethnic minorities in Jiangxi, Fujian, and Zhejiang provinces. Their presence is also reported in the provinces of Anhui and Guangdong. She people are shifting cultivation group in South China and had migrated from their primitive habitations in Fenghuang Mountain in Guangdong Chaozhou to Fujian, Zhejiang, Jiangxi, Anhui, and other provinces for more than a thousand years (Shi, 2013; Liu et al., 2017). The total population of the She group is 709,592 according to the 2010 census. Their presence in South Zhejiang is 25% and 53% in East Fujian. Their presence in Guangdong province is only 3.8% (27,000), although Guangdong Chaozhou Fenghuang Mountain is believed to be the definite headstream of She group’s culture and civilization (Liu et al., 2017). She people speak the She language with different dialects such as Shanke, Dongjia, and Huonie. She language is a branch of Sino-Tibetan language families and is the primary language for the She group (Lei, 2005). With reference to linguistics, the She language (mainly refers to Shanke and Dongjia dialects) belongs to the Hmong-Mien language branch (Lu and Wang, 2019). The Guangdong She population uses only the Huonie dialect. Interestingly, the divergences in languages may represent the differences in the genetic affinities of distinct She groups. Fujian She is more closely related to the Dongbei (Northern Han) and Hunan (Southern Han) populations (Liu et al., 2017). The genetic heterogeneities for the She population in the different regions indicate that the origins of Guangdong She and Fujian She may not be the same; Guangdong She may also not be a Hmong-Mien language-speaking group.

Y-chromosomal variant analysis for determining the patterns of present and past flow of genes between populations is very helpful (Oppenheimer, 2012). Y-chromosome short tandem repeats (Y-STRs) play an important role in forensic molecular biology (Prinz et al., 1997; Adnan et al., 2016). The use of Y-STRs also allows the simultaneous analysis of closely related and distantly related populations (Ballantyne and Kayser, 2012). Consequently, to address three main issues—(1) the forensic efficiencies of Promega PowerPlex^®^ Y23 System (Promega Corporation, Fitchburg, WI, United States) in Guangdong Hakka and Guangdong She groups, (2) the population structures of the two groups, and (3) the language classification of Guangdong She (Huonie Dialect)—we used the Promega 5-dye multiplex system to genotype 23 Y-STRs in 260 Guangdong Hakka and 215 Guangdong She males and evaluated the system effectiveness of forensic applications in two groups. Furthermore, we conducted population genetics employing diversified analyses among globally dispersed human populations and regional closely related populations to make the issues clearer.

## Materials and Methods

### Sample Preparation

In this study, a total of 475 unrelated male individuals (260 Hakka and 215 She) were recruited from Guangdong province of China (Figure 1C). Blood samples of all volunteers were collected using the FTA cards (Whatman^TM^, GE Healthcare, Chicago, IL, United States), with their written informed consent. This study and the procedures were approved by the Institutional Review Boards of Hainan Medical University and the Medical Ethics Committee of Hainan Medical University (no. HYLL-2020-012). All the experimental procedures were performed following the standards of the Declaration of Helsinki.

### Y-STR Amplification and Genotyping

One punched bloodstain paper (the size was 1.2 mm × 1.2 mm) was used as a PCR template directly for each sample without DNA extraction procedures. Y-STRs were co-amplified using Promega PowerPlex^®^ Y23 System (Promega Corporation, Fitchburg, WI, United States), which is a five-dye multiplex kit that analyzes the 17 Yfiler Y-STRs (DYS19, DYS385a/b, DYS389I, DYS389II, DYS390, DYS391, DYS392, DYS393, DYS437, DYS438, DYS439, DYS448, DYS456, DYS458, DYS635, and Y_GATA_H4) together with six discriminating Y-STRs (DYS481, DYS533, DYS549, DYS570, DYS576, and DYS643) on a Veriti^®^ 96 Well Thermal Cycler System (Thermo Fisher Scientific, Waltham, MA, United States) following the manufacturer’s instructions. The amplified products were separated by capillary electrophoresis on a 3500XL Genetic Analyzer (Thermo Fisher Scientific, Waltham, MA, United States), and data were analyzed using GeneMapper^®^ ID-X Software v1.6 (Thermo Fisher Scientific, Waltham, MA, United States).

### Statistical Analysis

Haplotype and allele frequencies were calculated using Arlequin v3.5 (Excoffier and Lischer, 2010). Gene diversity (GD) was calculated as per the following formula:

$$\text{HD}=\frac{n}{n-1}\,\left(1-\sum_{i}p_{i}^{2}\right)$$

where, *n* is the number of alleles at each Y-STR locus, and *P*_*i*_ is the frequency of the *i*-th allele. Haplotype diversity (HD) was estimated similarly as GD, while *n* and *P*_*i*_ were the total number of haplotypes and the frequency of the *i*-th haplotype, respectively. Discrimination capacity (DC) was determined as the ratio between the number of different haplotypes and the sample size. Match probability (MP) was defined as MP = $\sum P_{i}^{2}$, where *P*_*i*_ was the frequency of the *i*-th haplotype.

Genetic relationships between these populations and other reference populations were calculated employing *R*_*ST*_ (Excoffier et al., 1992; Excoffier and Smouse, 1994). Population pairwise genetic distances (*R*_*ST*_) and corresponding *p* values between different populations were estimated by analysis of molecular variance (AMOVA) by the online tool available at YHRD^1^. The principal component analysis (PCA) and multidimensional scaling plot (MDS) were conducted based on allele frequencies using the R programming language. Additionally, phylogenetic relationships among different populations were drawn in Molecular Evolutionary Genetics Analysis 7.0 software (Kumar et al., 2016) by neighbor-joining phylogenetic tree (Saitou and Nei, 1987) based upon genetic distance matrices (*R*_*ST*_ matrices) and visualized by the Interactive Tree of Life v5 (Letunic and Bork, 2019).

### Quality Control

The Y-STR typing experiments were performed strictly following the recommendations of the Chinese National Standards, Scientific Working Group on DNA Analysis Methods (SWGDAM, 2010) and the recommendations of the DNA Commission of the International Society of Forensic Genetics (Gusmao et al., 2006; Carracedo et al., 2013; Roewer et al., 2020). Control DNA 9948 and sdH_2_O were employed as positive and negative controls in each batch of PCR amplification and electrophoresis, respectively. In addition, our laboratory has passed the proficiency testing for Y-STR typing which is organized by YHRD and has been accredited under ISO/IEC 17025:2005 and China National Accreditation Service for Conformity Assessment.

The haplotype data of 475 unrelated male individuals from Guangdong Hakka and Guangdong She populations in the present study have been submitted to the YHRD database and received the accession number YA004707 (Guangdong Hakka, *n* = 260) and YA004708 (Guangdong She, *n* = 215). The Y-STR profiles, with variants (null alleles, off-ladder alleles, or copy number variants) for all samples, were re-amplified and re-genotyped by AmpFLSTR^®^ Yfiler^®^ PCR Amplification Kit (Thermo Fisher Scientific, Waltham, MA, United States) and Geno-ID Y41 Human Typing (Guangzhou Koalson Intelligent BioRobotics, Guangzhou, Guangdong, China) for confirmation.

## Results and Discussion

### Allele Frequencies, Allele Numbers, and GD Values

The allele frequencies, allele numbers, and GD values of 23 Y-STR loci in the Guangdong Hakka and She groups are presented in Supplementary Tables 1, 2, respectively.

In Guangdong Hakka, 176 distinct alleles were observed at all loci, and the corresponding allelic frequencies ranged from 0.0038 to 0.7423 (DYS438). The number of different alleles varied from three (DYS437) to 40 (DYS385a/b). The GD values of six Y-STRs were greater than 0.9, and the highest (0.9671) and lowest (0.4877) estimates of GD corresponded to loci DYS385a/b and DYS438, respectively. We identified three off-ladders at DYS576 (19.1) and DYS458 (16.1) and the microvariant 19.1 at DYS576 which happened twice. All loci having intermediate values found in the Guangdong Hakka were re-amplified and re-genotyped for confirmation and were commonly observed in YHRD.

In the Guangdong She group, a total of 155 different alleles were obtained, and the number of diverse alleles ranged from four (at seven Y-STRs) to 36 (at DYS385a/b). The allele frequencies varied from 0.0047 to 0.8605 (DYS438). On one hand, the set of 23 Y-STR loci had a high level of genetic polymorphisms in the She group, DYS458 (0.9526), DYS385a/b (0.9180), DYS481 (0.9146), and DYS448 (0.9060), while the DYS438 (0.3277) and DYS391 (0.4875) showed extreme values of the GD distribution (with GD values < 0.5). In this study, no null allele, off-ladder, and copy number variant were found in the Guangdong She group.

### Haplotypes and Forensic Parameters

The haplotypes and haplotype frequencies of 23 Y-STRs in the two groups are shown in Supplementary Tables 3, 4, respectively. A total of 231 different haplotypes were found in 260 Guangdong Hakka male individuals, of which 208 (90.04%) were unique. It was observed that 18 haplotypes (H006–H023) occurred twice, four haplotypes (H002–H005) occurred thrice, while only one haplotype (H001) was observed as being shared by four individuals (Supplementary Table 3). Genotyping with the 23 Y-STRs determined 122 distinct haplotypes in the Guangdong She group, and the fraction of unique haplotypes was only 53.28%. Additionally, 43 haplotypes (S015–S057) were detected twice, six haplotypes (S009–S014) and five haplotypes (S004–S008) were found three and four times, respectively, and the haplotypes S003, S002, S001 appeared five, six, and 15 times, respectively (Supplementary Table 4).

As shown in Table 1, the overall HD values of the Guangdong Hakka and She groups were 0.9994 and 0.9939, respectively. Moreover, it can be seen from Table 1 that the DC value of the Guangdong She group (0.5674) was much lower than those of the Guangdong Hakka group (0.8885) and Guangdong Han group (0.9706), which demonstrated that the discrimination power of Promega PowerPlex^®^ Y23 System was not seemingly appropriate for the Guangdong She group. With the Guangdong She group being relatively isolated, there was limited gene flow, which led to the decrease in the discrimination capacity of the Y-STR system. In this regard, there is a dire need to get more knowledge about the mutability of currently known Y-STRs and to incorporate the rapidly mutating Y-STRs into forensic systems to enhance the capacity of discrimination, especially for isolated groups.

**TABLE 1:** Forensic characteristics of 23 Y-STRs in Guangdong populations (Hakka, She, and Han).

Number of observed haplotypes	She	Hakka	Han^a^
1	65	208	320
2	43	18	10
3	6	4	
4	5	1	
5	1		
6	1		
15	1		
*N*	122	231	330
FUH	0.5328	0.9004	0.9697
HD	0.9939	0.9994	0.9997
DC	0.5674	0.8885	0.9706
MP	1.42E-02	4.91E-03	3.31E-03

*^*a*^Unpublished data for Guangdong Han (*n* = 340), which genotyped 23 Y-STRs by Promega PowerPlex^®^ Y23 System.*

**N*, number of haplotypes; FUH, fraction of unique haplotypes; HD, haplotype diversity; DC, discrimination capacity; MP, match probability.*

### PCA

Dimensionality reduction analyses including PCA, MDS, linear discriminant analysis, Laplacian Eigenmaps, and locally linear embedding can accelerate the speed of algorithm execution, improve the performance of the analysis model, and reduce the complexity of data at the same time. To illustrate the genetic landscapes of globally dispersed human populations, especially for Guangdong Hakka and She groups, the dimensionality reduction analyses (PCA and MDS) were conducted based on the frequencies of 23 Y-STRs.

**FIGURE 2:**
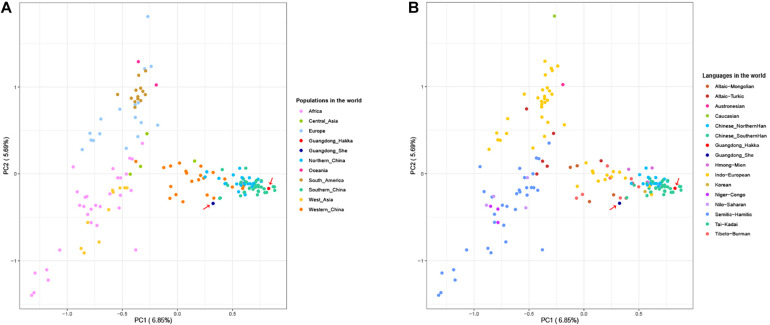
Principal component analysis (PCA) based on the frequencies of 23 Y-STRs among Guangdong Hakka, Guangdong She, and 159 worldwide human populations. **(A)** PCA from geographical scale. **(B)** PCA from linguistic scale.

The PCA provides a method of visualizing the essential patterns of genetic relationships and allows us to identify and plot the major patterns within a multivariate dataset to indicate that the populations with closer geographical distances have more intimate relationships. We collected the 23 Y-STR frequency profiles of 48,162 individuals from 159 worldwide populations having nine language families within five continents (Supplementary Table 5) to conduct PCA with Guangdong Hakka and She groups (Yaju et al., 2014; Westen et al., 2015; Bai et al., 2016b; Garcia et al., 2016; Jung et al., 2016; Pickrahn et al., 2016; Wang L. et al., 2016; Wang Y. et al., 2016; Guo et al., 2017; Iacovacci et al., 2017; Jun et al., 2017; Lacerenza et al., 2017; Qiang et al., 2017; Spolnicka et al., 2017; Wang M. et al., 2017; Zgonjanin et al., 2017; Zhang et al., 2017; Cao et al., 2018; Fan et al., 2018a,b,c; Khubrani et al., 2018; Liu Y. et al., 2018; Juan et al., 2018; Liu Y. J. et al., 2018; Wang et al., 2018, 2020; Zhou et al., 2018; D’Atanasio et al., 2019; Fan et al., 2019; He et al., 2019; Henry et al., 2019; Ip et al., 2019; Jankova et al., 2019; Kai et al., 2019; Lang et al., 2019; Liu et al., 2019, 2020; Luo et al., 2019; Santos Stange et al., 2019; Tao et al., 2019a,b; Wang C. Z. et al., 2019; Wang X. et al., 2019; Wang Y. et al., 2019; Wu et al., 2019; Xie et al., 2019; Yaju et al., 2019, 2020; Zhabagin et al., 2019; Zhang et al., 2019; Al-Snan et al., 2020; Dezhi et al., 2020; Fan et al., 2020; Feng et al., 2020; Haiying et al., 2020; Hanguang et al., 2020; Jannuzzi et al., 2020; Kang et al., 2020; Shonhai et al., 2020; Song et al., 2020; Tang et al., 2020; Yin et al., 2020; Zeyad et al., 2020). As shown in Figure 2, the first and second components (PC1 and PC2) accounted for 6.85 and 5.69% of the total variances observed within these populations, respectively. From the perspective of geography, Guangdong Hakka was located in the cluster of south Chinese populations (Figure 2A). Additionally, Guangdong Hakka was observed close to the Southern Han populations (Figure 2B) from the linguistic point of view, while the Guangdong She group was situated in a relatively isolated location with no definite relationship with other Chinese populations from both geographic and linguistic scales (Figure 2).

**FIGURE 3:**
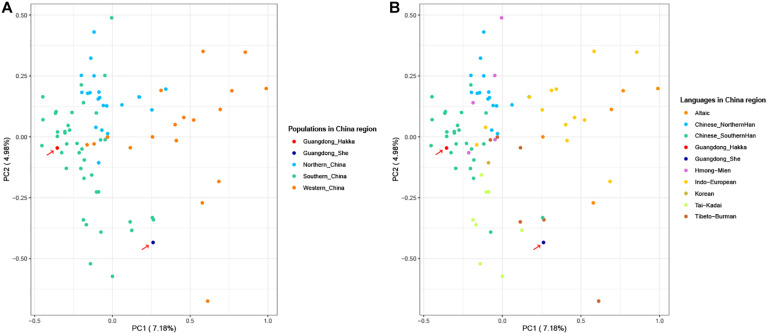
Principal component analysis (PCA) based on the 23 Y-STRs frequency profiles among Guangdong Hakka, Guangdong She, and 83 Chinese populations. **(A)** PCA from geographical scale. **(B)** PCA from linguistic scale.

To further clarify the genetic relationships on a relatively small scale, we performed PCA within Chinese populations (Figure 3). Populations from different regions of China clustered separately, and Guangdong Hakka and Southern Han clustered similarly on the left side. Guangdong She distributed on the middle bottom and clustered with south Chinese populations. However, from the perspective of linguistics, the language relationships between Guangdong She and the surrounding south Chinese populations which are illustrated in Figure 3B were still not distinct.

### MDS

**FIGURE 4:**
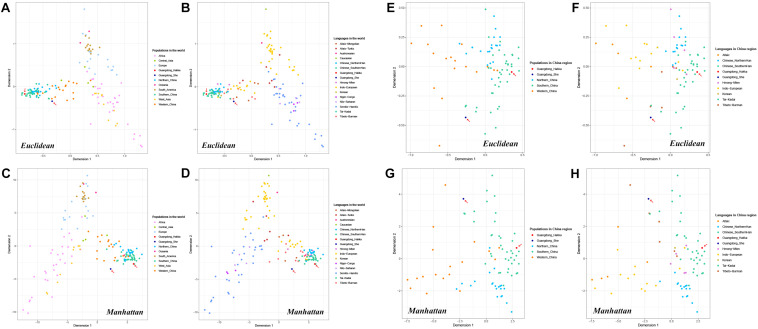
Multidimensional scaling (MDS) plots based on the frequencies of 23 Y-STRs among Guangdong Hakka, Guangdong She, and 159 worldwide human populations (including 83 Chinese populations). **(A)** Euclidean-based MDS plot in worldwide populations from geographical scale. **(B)** Euclidean-based MDS plot in worldwide populations from linguistic scale. **(C)** Manhattan-based MDS plot in worldwide populations from geographical scale. **(D)** Manhattan-based MDS plot in worldwide populations from linguistic scale. **(E)** Euclidean-based MDS plot in Chinese populations from geographical scale. **(F)** Euclidean-based MDS plot in Chinese populations from linguistic scale. **(G)** Manhattan-based MDS plot in Chinese populations from geographical scale. **(H)** Manhattan-based MDS plot in Chinese populations from the linguistic scale.

MDS plots were conducted based on allele frequencies by Euclidean distance and Manhattan distance, respectively. As shown in Figure 4, each population was represented by a small dot with a different color in the multidimensional space, and the distances between the small dots showed the genetic relationships among the populations in distinct geographic areas or with different language families.

Whether from the perspective of geographical or linguistic scale, the results of MDS analysis by Euclidean distance (Figures 4A,B) had no apparent differences with the principal component analysis (Figures 2A,B). Moreover, in the Manhattan distance-based MDS plots (Figures 4C,D), Guangdong Hakka intertwining with the Southern Han cluster was in accordance with the performances in the MDS conducted by Euclidean distance (Figures 4A,B) and PCA plots (Figure 2). In addition, Guangdong She was located in a relatively isolated place.

Furthermore, to illustrate partial genetic relationships, we collected Guangdong Hakka, Guangdong She, and 83 other Chinese populations to perform MDS analyses with Euclidean distance and Manhattan distance (Figures 4E–H). The MDS results demonstrated that Guangdong Hakka had a close relationship with Southern Han populations, and Guangdong She was situated in a relatively isolated location from the perspectives of geography and linguistics, which were in line with the results of the PCA.

### R_*ST*_ and Phylogenetic Analyses

**FIGURE 5:**
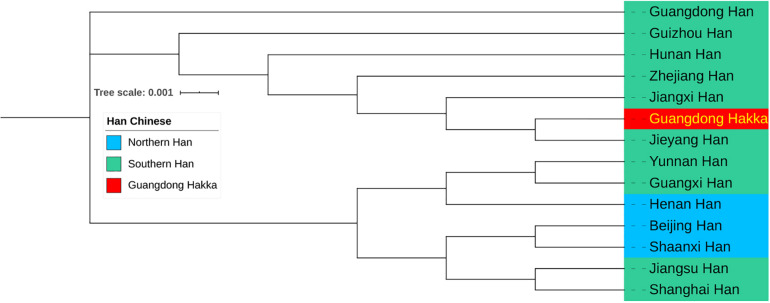
Neighbor-joining phylogenetic tree between Guangdong Hakka and 13 Han Chinese populations (including three Northern Han and 10 Southern Han) based on the matrix of pairwise *R*_*ST*_ values.

The results of the calculations of pairwise *R*_*ST*_ and the corresponding *p* values between Guangdong Hakka and 13 other Chinese Han populations from Southern and Northern Han regions (Kwak et al., 2005; Wu et al., 2011; Zhang et al., 2014; Shi et al., 2015; Shu et al., 2015; Bai et al., 2016b; Li et al., 2016; Wang L. et al., 2016; Wang Y. et al., 2016; Zhou et al., 2016, 2018; Nothnagel et al., 2017; Wang H. et al., 2017; Huang et al., 2019; Lang et al., 2019; Sun et al., 2019; Guan et al., 2020) based on 23 Y-STR haplotypes are listed in Supplementary Table 6. The population Guangdong Hakka has been found to be closely related to Guangdong Jieyang Han (*R*_*ST*_ = 0.0028) and Jiangxi Han (*R*_*ST*_ = 0.0029). Subsequently, the results of the phylogenetic analysis among Guangdong Hakka and Chinese Han populations are displayed in a phylogenetic tree based on the neighbor-joining method (Figure 5). Guangdong Hakka was first clustered with Guangdong Jieyang Han, followed by Jiangxi Han. Geographically, Jieyang is surrounded by Shanwei, Chaozhou, and Meizhou, whereas the habitations for Guangdong Hakka (Figure 1C) and Jiangxi bordering with Guangdong in the southwest were the dominating Hakka settlements. The phylogenetic tree showed the relationships between Guangdong Hakka and other Han populations in genetics, which were in accordance with the geographical relations. The paternal relationships revealed by Y-STRs indicate that Guangdong Hakka had close relationships with Southern Han, and there were extensive gene flows between Guangdong Hakka and the surrounding Han populations. Our results corroborated the findings of previous studies (Du et al., 2019; Han et al., 2020), which were conducted by using STRs and Y-STRs on the Guangdong Meizhou Hakka population.

**FIGURE 6:**
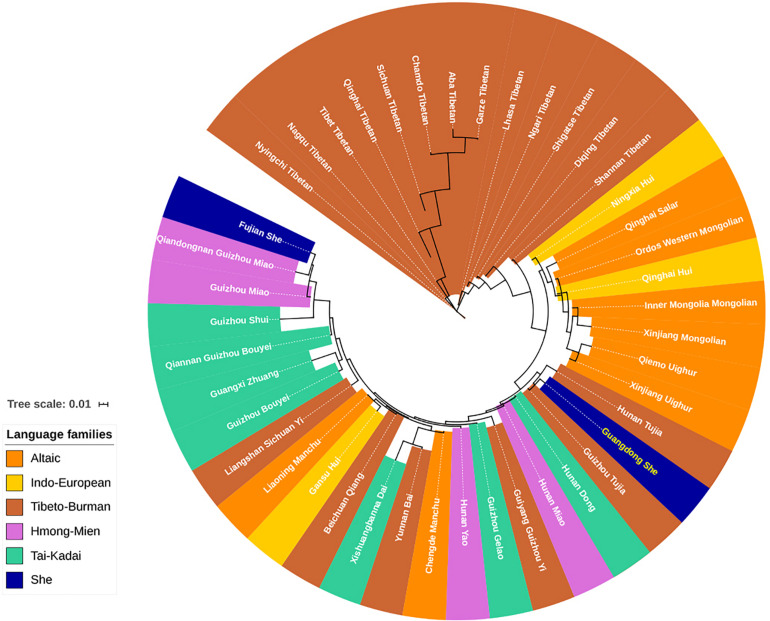
Neighbor-joining phylogenetic tree between Guangdong She and 42 other minorities with five different language families based on pairwise *R*_*ST*_ values.

From the dimensionality reduction analyses discussed above, the genetic relationships between Guangdong She and other Chinese populations were not clarified. In addition, we employed the Y-STR haplotype profiles of 42 Chinese minorities and Guangdong She to assess the population pairwise genetic distances by AMOVA (Zhu et al., 2005, 2006a,b, 2008; He and Guo, 2013; Shan et al., 2014; Luo et al., 2015, 2019; Ou et al., 2015; Shu et al., 2015; Bai et al., 2016a; Bian et al., 2016; Fu et al., 2016; Gao et al., 2016; Yao et al., 2016; Guo et al., 2017; Ji et al., 2017; Zhao et al., 2017; Cao et al., 2018; Chen et al., 2018; Liu Y. J. et al., 2018; Atif et al., 2019; Liu et al., 2019; Xie et al., 2019; Dezhi et al., 2020; Feng et al., 2020; Song et al., 2020; Tang et al., 2020). The pairwise *R*_*ST*_ and the corresponding *p* values between Guangdong She and Chinese minority populations from five linguistically different families are displayed in Supplementary Table 7. No differences were observed between Guangdong She and Guizhou Tujia populations (*R*_*ST*_ = 0.0046, *p* = 0.0574), while significant genetic differences were observed between Guangdong She and all other Chinese minority populations (*p* < 0.05). The phylogenetic relationships between Guangdong She and 42 Chinese minorities were visualized in the neighbor-joining tree. As shown in Figure 6, the Tibeto-Burman language-speaking Tibetans and Altaic language-speaking Uighurs and Mongolians clustered together at the upper end, while the language relationships at the bottom, especially for Tai-Kadai, Hmong-Mien, and Tibeto-Burman, were ambiguous. The Guangdong She group clustered with two Tibeto-Burman populations, Guizhou Tujia and Hunan Tujia. However, the Fujian She group congregated with Guizhou Hmong-Mien language-speaking Miao populations.

### Prediction of Y-Haplogroups

**FIGURE 7:**
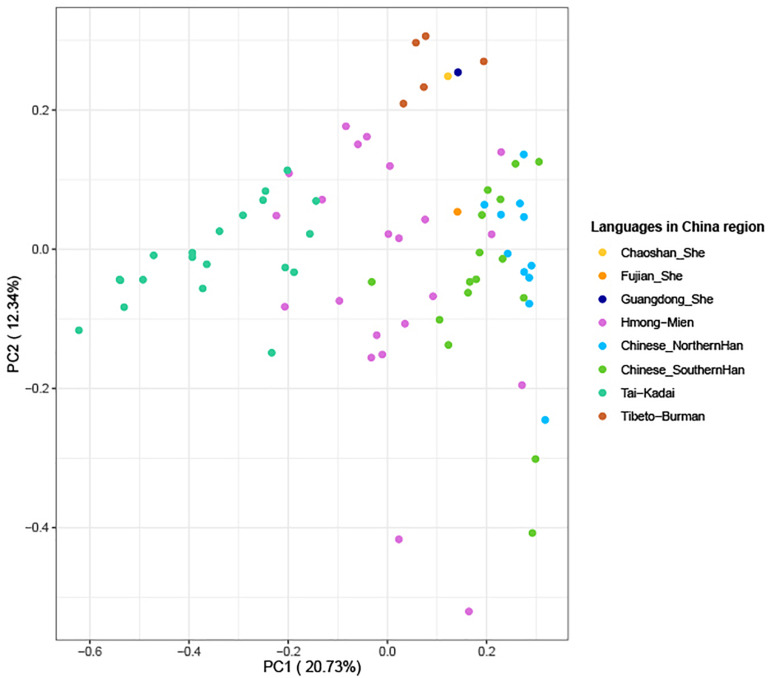
Principal component analysis based on Y-haplogroup frequencies between three She groups and 74 populations (4,195 individuals in total, which included 22 Hmong-Mien, 20 Tai-Kadai, 16 Southern Han, 11 Northern Han, and five Tibeto-Burman language-speaking populations mainly from Southeast and East Asia).

The above-mentioned phylogenetic analysis hinted that Guangdong She clustered with Tibeto-Burman language-speaking Tujia populations in the same branch. To make further confirmation for the genetic relationships, we employed our in-house database which was composed of 37,754 pieces of Y-SNP/STR data and 109,142 Y-STR (Wang et al., 2015) mainly from East and Southeast Asia to make more precise predictions for 215 Guangdong She males in this study. Finally, 212 out of the 215 genotyped Y-STRs (98.60%) were observed, and a total of six Y-haplogroups were defined in Guangdong She which belong to major clades O2 and O1. The predominantly detected haplogroups were O2-M122 (45.75%), O2a2a1a2-M7 (25.47%), O1a-M119 (10.38%), O1b1a1a-M95 (8.02%), O-M175 (7.08%), and O2a2b1a1-M117 (3.30%), which were determined according to ISOGG, 2019^2^. In addition, a PCA graph was performed among 77 populations (Wen et al., 2004; Gan et al., 2008; Li et al., 2008; Cai et al., 2011; Deng et al., 2013; Fan et al., 2018c; Rowold et al., 2019; Luo et al., 2020; Fan et al., 2021), which were composed of 4,195 individuals in total, including Tai-Kadai, Hmong-Mien, Tibeto-Burman, and Chinese (Southern and Northern Han) populations from Southeast and East Asia, which included three She groups (Fujian, Guangdong Chaoshan, and Guangdong She). From the diagram in Figure 7, the first and second principal components could explain 33.07% of the total variances. Moreover, the populations with different language branches were separated relatively, and the Guangdong She group had a close relationship with the Guangdong Chaoshan She group and clustered with Tibeto-Burman language-speaking populations (including Tujia, Tibetan, and Naxi minorities), while Fujian She located between Hmong-Mien and Han populations, especially Southern Han. The interpopulation comparison demonstrated that (1) different branches of She populations and Fujian and Guangdong She groups may have distinct origins from the perspectives of genetics and linguistics, especially from phylogenetic analyses based on Y-STRs and Y-SNPs, and (2) Guangdong She and Chaoshan She groups have close affinities with Tibeto-Burman language-speaking populations based on the evidence of Y-haplogroups which contained information about the subsequent colonization, differentiations, and migrations overlaid on recent population ranges.

## Conclusion

In the present study, a total of 475 unrelated Guangdong males (260 Hakka and 215 She) were genotyped by using 23 Y-STRs (including 17 Yfiler and 6 additional Y-STRs) by Promega PowerPlex^®^ Y23 System. For Guangdong Hakka, a total of 176 different alleles were found, with corresponding allelic frequencies ranging from 0.0038 to 0.7423 and GD values that varied from 0.4877 to 0.9671, and the systematic effectiveness for Guangdong Hakka was observed to be sufficient (HD = 0.9994, DC = 0.8885). From the perspectives of geographical and linguistic scales, the phylogenetic analyses indicated that Guangdong Hakka had a close relationship with Southern Han, and there were extensive gene flows between Guangdong Hakka and the surrounding Han populations. For Guangdong She, we identified 155 distinct alleles with a range of allele frequencies from 0.0047 to 0.8605. The GD values for 23 Y-STRs ranged from 0.3277 to 0.9526, and the overall DC and HD were 0.5674 and 0.9939, respectively. The predominant haplogroups of the Guangdong She group were O2-M122 and O2a2a1a2-M7. Moreover, Guangdong She clustered with Tibeto-Burman language-speaking populations (Guizhou Tujia and Hunan Tujia), which demonstrates that the Guangdong She group seems to be one branch of Tibeto-Burman populations and the Huonie dialect of She languages may be a branch of Tibeto-Burman language families.

## Data Availability Statement

The datasets presented in this study can be found in online repositories. The names of the repository/repositories and accession number(s) can be found in the article/Supplementary Material.

## Ethics Statement

The studies involving human participants were reviewed and approved by Medical Ethics Committee of Hainan Medical University. The patients/participants provided their written informed consent to participate in this study.

## Author Contributions

HF contributed to conceptualization, formal analysis, visualization, and writing—original draft preparation. CL and DP took charge of resources and contributed to project administration. HF, LW, and KR were in charge of software. LD, YL, QX, and YZ conducted the investigation. LD, YZ, FW, and ZD performed the validation. CL contributed to data curation. QX, SN, and MJ contributed to writing—review and editing. PQ and S-QW supervised the study. CL, HF, FW, and ZD took charge of funding acquisition. All authors reviewed the manuscript.

## Conflict of Interest

The authors declare that the research was conducted in the absence of any commercial or financial relationships that could be construed as a potential conflict of interest. The handling editor AA declared a past collaboration with the author SN.

## Footnotes

1 https://yhrd.org/

2 https://isogg.org/tree/
